# Ecotoxicological Insights From Ex Vivo Exposure of Whole Blood to PFOS and Glyphosate: Oxidative Stress and Immune Disruption

**DOI:** 10.1002/tox.70053

**Published:** 2026-02-23

**Authors:** Francesco Molinari, Gianluca Antonio Franco, Francesca Inferrera, Nicla Tranchida, Antonella Iaconis, Maria Rizzo, Giuseppe Piccione, Enrico Gugliandolo, Davide Di Paola

**Affiliations:** ^1^ Department of Veterinary Sciences University of Messina Messina Italy; ^2^ Department CHIBIOFARAM University of Messina Messina Italy

## Abstract

This study investigated the immunomodulatory effects of PFOS and glyphosate (GLY), both individually and in combination, on the whole blood of three ruminant species (cow, goats, and sheep) exposed ex vivo to environmentally relevant concentrations. The research focused on key biomarkers of oxidative stress (MDA), inflammation (myeloperoxidase activity, nitrite production), and immune function (TNFα and IL‐6 cytokine production, both at baseline and following LPS stimulation). Results indicated species‐specific differences in oxidative stress response, with sheep and goats showing higher MDA levels under combined PFOS and GLY exposure. Furthermore, co‐exposure exacerbated inflammatory responses (MDA activity and NO2‐ production) in LPS‐stimulated sheep and goats. Critically, a synergistic effect of PFOS and GLY co‐exposure significantly increased both TNFα and IL‐6 production across all species, even without LPS stimulation, suggesting a heightened pro‐inflammatory state. These findings raise concerns about the potential of these contaminants to disrupt immune homeostasis in grazing animals, impacting their health, productivity, and disease resistance, and potentially posing risks to human health through food chain contamination. The study highlights the need for further research to elucidate the underlying molecular mechanisms and assess the long‐term effects of chronic exposure to these contaminants on animal health and potential human health risks.

## 1 Introduction

Endocrine disruptors are chemicals, both synthetic and natural, that interfere with our endocrine system [1]. The endocrine system is a complex system of glands manufacturing hormones, which are very important in regulating growth, reproduction, and metabolism [2]. Endocrine disruptors can mimic hormones, block their action, or alter their production, thereby contributing to a wide range of health problems. These chemicals are pervasive in the environment, being detected in food, water, air, household items, and agricultural systems [3]. Among the most significant are perfluorooctane sulfonic acid (PFOS) and glyphosate (GLY) [4, 5].

PFOS have been extensively used in waterproof and stain‐resistant textiles, firefighting foams, and numerous industrial products. Due to their persistence in both the environment and the human body, they readily contaminate soil and aquatic systems once released [6, 7]. PFOS can be absorbed by plants, ingested by grazing animals, and subsequently biomagnified through the food chain, reaching higher concentrations in predatory fish and ultimately in humans [8]. Similarly, GLY, one of the most widely applied pesticides worldwide and the active ingredient in Roundup, readily infiltrates soil and contaminates surface and groundwater after agricultural application [9]. Plants absorb it via their roots, accumulating it in their tissues, and grazing animals feeding on these contaminated plants ingest the pesticide, which can accumulate in their fatty tissues [10, 11]. This process contributes to bioaccumulation across the food chain.

Grazing animals such as goats, sheep, and cows provide nutritious food while playing a fundamental role in maintaining biodiversity and sustaining healthy ecosystems [12]. Goats, through their browsing behavior, act as natural “gardeners,” even consuming tough plant species and reducing fire risk [13]. Sheep contribute by selectively feeding on weeds, which helps maintain pasture balance [14, 15]. Cattle, through their weight, compact soil and stimulate deeper root growth, thereby enriching soil fertility [16]. Beyond these agronomic benefits, grazing animals act as sentinel species: contaminants can be traced from their hair, hooves, and feces, providing valuable indicators of soil and water pollution with direct relevance to environmental health [17]. In addition, their grazing activity promotes biodiversity, creating habitats for insects, birds, and small mammals.

The present study was conducted to examine the in vitro effects of PFOS and GLY on whole blood from sheep, goats, and cows. Specifically, we aimed to evaluate how these substances modulate the immune response of these animals to an inflammatory stimulus, as induced by lipopolysaccharide (LPS). We hypothesized that combined exposure to PFOS and GLY would induce a synergistic pro‐inflammatory response in ruminant whole blood, exceeding the effects of either compound alone, and that this effect would be species‐specific. Changes in various immunological parameters were analyzed to elucidate the mechanisms underlying the interactions of these environmental contaminants with the ruminant immune system.

## 2 Materials and Methods

### 2.1 Animals and Blood Collection

Blood samples were collected from 60 healthy animals, including 20 cows, 20 goats, and 20 sheep. From all animals, blood samples were collected by jugular venipuncture into an 8 mL heparinized vacutainer tube. All samples were taken with warning at 8:00 AM. Immediately after collection, blood samples were placed in refrigerated bags and transported to the laboratory for the analysis. Animal husbandry and experimentation protocols adhered to the guidelines set forth by the Guide for the Care and Use of Laboratory Animals and Directive 2010/63/EU.

### 2.2 Whole Blood Culture and Stimulation

Whole blood cultures and stimulations were conducted as previously described [18]. Briefly, blood from each subject was diluted 1:2 with RPMI 1640 culture medium (Gibco, Life Technologies Corporation, Waltham, MA, USA) supplemented with 200 U/mL penicillin and 200 mg/mL streptomycin. 500 μL aliquots of diluted whole blood from each subject were incubated at 37°C with 5% CO2 for 24 h. Incubation was performed with or without the following: heptadecafluorooctansulforic acid solution (Sigma Aldrich, 77 283), GLY (MedChemExpress, Cat.# HY‐B0863), LPS (ChemCruz, Lypopolysaccharide, E. coli O55:B5), and combinations thereof:

1. Control: whole blood;
2. LPS 10 μg/mL: whole blood + LPS 10 μg/mL;
3. PFOS 10 μM: whole blood + PFOS 10 μM;
4. GLY 100 μM: whole blood + GLY 100 μM;
5. PFOS+GLY 1:1: whole blood + PFOS 10 μM + GLY 100 μM;
6. PFOS+LPS: whole blood + LPS 10 μg/mL + PFOS 10 μM;
7. GLY + LPS: whole blood + LPS 10 μg/mL + GLY 100 μM;
8. PFOS+GLY + LPS 1:1: whole blood + LPS 10 μg/mL + PFOS 10 μM + GLY 100 μM;

Doses for PFOS and GLY were chosen based on bibliography [19, 20, 21, 22, 23]. After incubation, samples were centrifuged at 2000× *g* for 5 min at room temperature for each condition. In particular, the supernatants and cell pellet were harvested and stored immediately at −20°C until further use.

### 2.3 Nitrite Evaluation

To determine nitrite levels, the Griess reaction was performed on the supernatant. Specifically, 50 μL of supernatant was added to a 96‐well plate, followed by the addition of 50 μL of 1% sulfanilamide solution in 5% phosphoric acid [24]. After a 10‐min incubation, 50 μL of 0.1% NED solution was added to each well, and the plate was incubated for an additional 10 min. Absorbance was measured at 540 nm within 30 min using a plate reader [25, 26, 27].

### 2.4 MDA

To determine lipid peroxidation, MDA production was measured using the thiobarbituric acid assay (TBARS). The plasma was subjected to the TBARS assay, as previously described, to assess MDA levels, an indicator of lipid peroxidation [25, 26].

### 2.5 Cytokines Evaluations

Using enzyme‐linked immunosorbent assay (ELISA) kits, serum levels of IL‐1β (CUSABIO Cat. CSB‐E12986B for cattle, CSB‐E10115Sh for sheep, CSB‐E16568G for goat) and IL‐6 (CUSABIO Cat. CSB‐E12899B for cattle, CSB‐E10116Sh for sheep, CSB‐E14360G for goat) were measured in accordance with the manufacturer's instructions [28, 29, 30]. Intra‐assay variation was assessed by analyzing the positive control 3 times within the same plate, providing a measure of ELISA repeatability under identical conditions. To evaluate inter‐assay variation, the positive control was tested in 3 replicates across five different plates on five separate days. A coefficient of variation (CV) below 15% was considered acceptable [29, 31].

### 2.6 Statistical Analysis

Each experiment included three independent replicates. The data resulting from all experiments are expressed as means ± SEM. Statistical differences between groups were compared using one‐way or two‐way ANOVA, followed by Bonferroni's post hoc test for multiple comparisons, using GraphPad Prism version 10 (GraphPad Software Inc., La Jolla, CA, USA). A *p*‐value of less than 0.05 was considered statistically significant [30].

## 3 Results

### 3.1 Ex Vivo Exposure to Contaminants and Mixtures

**FIGURE 1:**
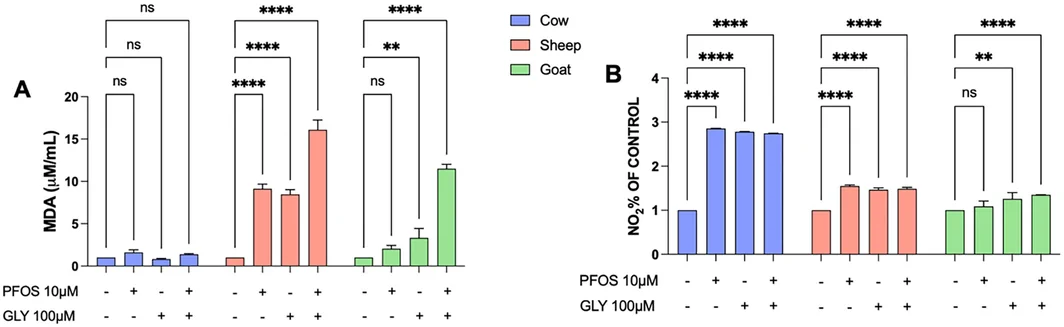
Graphs representing the results obtained in the assessment of oxidative stress by assay of MDA (A), and nitrite (B). Data are representative of at least three experiments, means ± SEM. ns = not significant; **p value < 0.01; ***p value < 0.001; ****p value < 0.0001.

To assess the impact of the contaminants PFOS (10 μM) and GLY (100 μM), alone or in combination, on oxidative stress, malondialdehyde (MDA) and nitrite (NO_2_^−^) levels were measured in bovine, ovine, and caprine samples. As shown in Figure 1A, cow samples displayed consistently low MDA levels across all conditions, with no significant differences among treatments (ns). In sheep, PFOS or GLY exposure significantly elevated MDA compared to control (*p* < 0.0001), and the combined PFOS + GLY exposure induced the highest levels of lipid peroxidation (*p* < 0.0001 vs. control). Similarly, goat samples showed a significant increase in MDA upon PFOS exposure (*p* < 0.0001), which was further enhanced by co‐exposure to GLY (*p* < 0.01). As shown in Figure 1B, cows exhibited a marked increase in NO_2_^−^ levels following PFOS exposure (~3‐fold vs. control, *p* < 0.0001). GLY alone also significantly elevated NO_2_^−^ (*p* < 0.0001), but combined PFOS + GLY exposure did not further amplify the effect. In sheep, both PFOS and GLY individually increased NO_2_^−^ (*p* < 0.0001), while co‐exposure did not cause additive effects. In goats, PFOS or GLY exposure had no significant effect compared to control (ns). Overall, these findings reveal species‐specific susceptibilities to the two contaminants. Sheep and goats were highly sensitive to PFOS‐ and GLY‐induced lipid peroxidation, while cows primarily exhibited nitrosative stress responses. Co‐exposure often exacerbated oxidative stress, particularly in sheep and goats.

To assess whether the alteration caused by exposure to PFOS and GLY could alter the response to an inflammatory stimulus, plasma from grazing animals was stimulated with LPS and endocrine disruptors.

**FIGURE 2:**
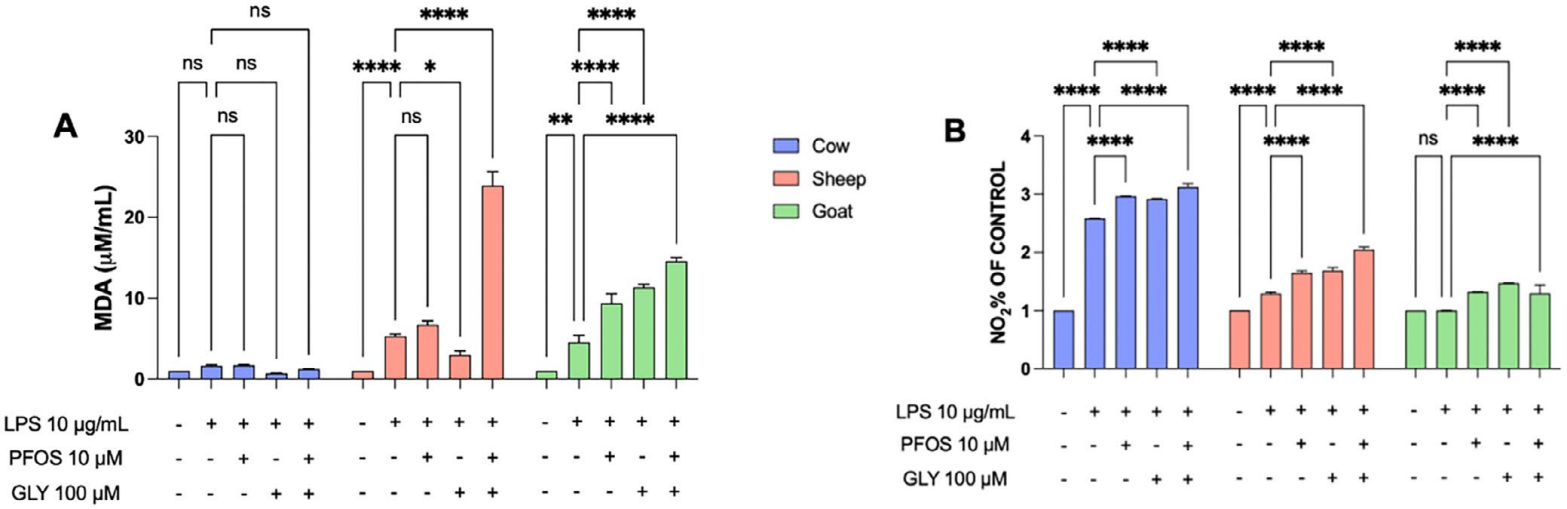
Graphs representing the results obtained in the assessment of oxidative stress by assay MDA (A), and nitrite (B). Data are representative of at least three experiments, means ± SEM. ns = not significant; *p value < 0.05; ** < *p* value < 0.01; ***p value < 0.001; ****p value < 0.0001.

As shown in Figure 2A, cattle samples again showed consistently low MDA levels across all treatments, with no significant changes (ns). In sheep, exposure to PFOS or GLY significantly increased MDA compared to LPS control (*p* < 0.0001), and co‐exposure to PFOS + GLY further enhanced lipid peroxidation (*p* < 0.05). Similarly, goat samples showed a significant rise in MDA following PFOS or GLY exposure (*p* < 0.01 to *p* < 0.0001), with the combined treatment producing the highest levels. Compared to the previous experiment without LPS, the overall magnitude of MDA induction was higher, suggesting that LPS acts synergistically with PFOS and GLY to amplify lipid peroxidation in sheep and goats. As shown in Figure 2B, cattle displayed a strong increase in NO_2_^−^ levels upon LPS stimulation alone. Additional exposure to PFOS or GLY further elevated nitrosative stress compared to LPS control (*p* < 0.0001), but no additive effect was observed with the PFOS + GLY combination. In sheep, LPS alone induced modest NO_2_^−^ production, which was significantly augmented by PFOS or GLY (*p* < 0.0001). The combined exposure caused further elevation, indicating additive effects of the contaminants in an inflammatory context. In goats, LPS did not markedly increase NO_2_^−^ compared to control (ns), but both PFOS and GLY significantly elevated levels when combined with LPS (*p* < 0.0001), highlighting a sensitizing effect of contaminants that was not observed in the absence of LPS. Overall, these results show that the presence of LPS potentiates the oxidative and nitrosative stress responses induced by PFOS and GLY. While cows primarily respond with nitrosative stress and sheep and goats with lipid peroxidation, the inflammatory stimulus broadens susceptibility, particularly in goats, where contaminants exacerbate stress responses only under LPS challenge.

### 3.2 Effect on TNFα and IL‐6 Release

**FIGURE 3:**
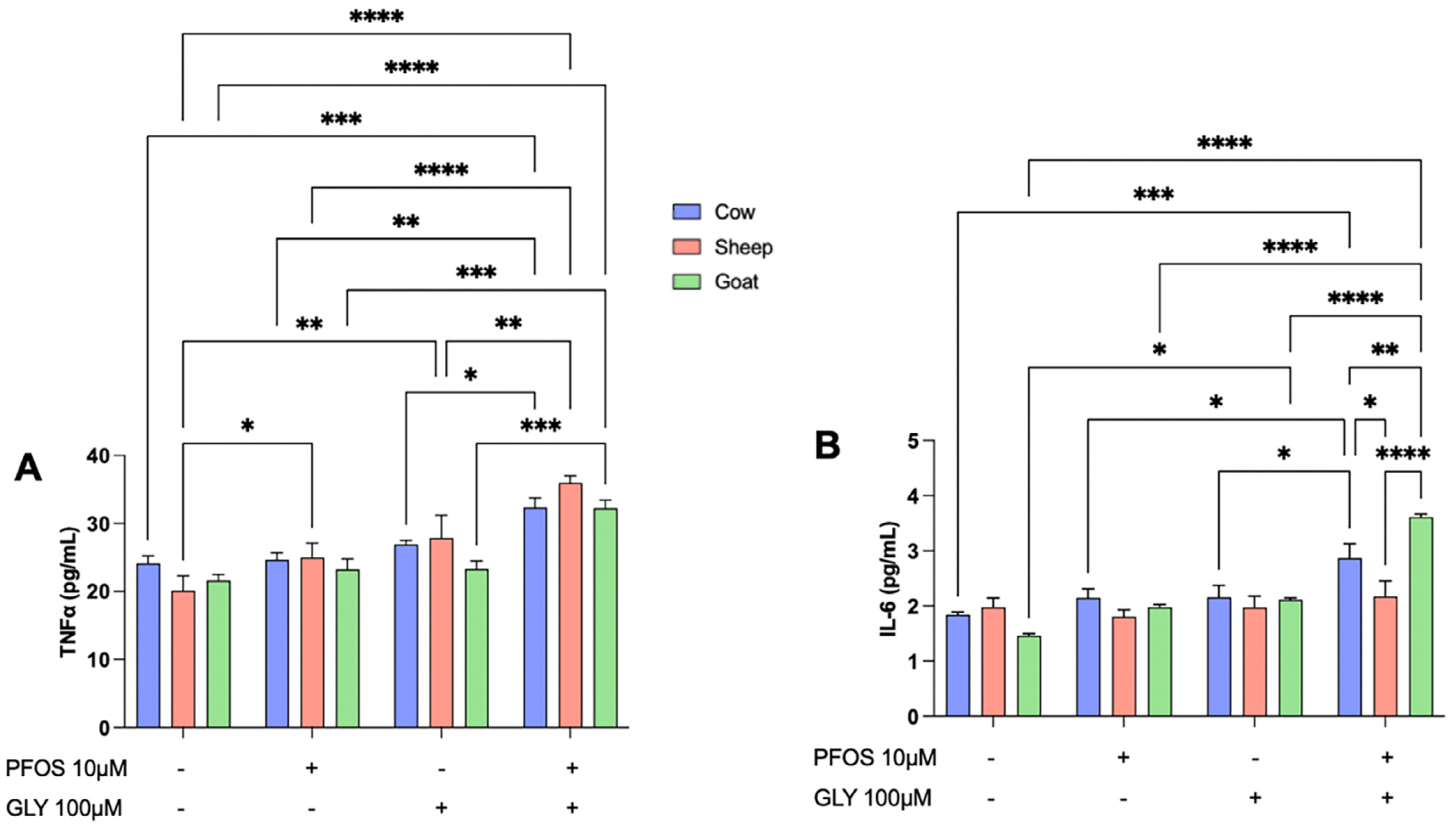
Graphs representing the results obtained in the evaluation of pro‐inflammatory cytokines TNF‐α (A) and IL‐6 (B). Data are representative of at least three experiments, means ± SEM. *p value < 0.05; **p value < 0.01; ***p value < 0.001; ****p value < 0.0001.

To assess the effects of PFOS (10 μM) and GLY (100 μM) on proinflammatory cytokine production, TNF‐α (Figure 3A) and IL‐6 (Figure 3B) levels were measured in bovine, ovine, and caprine cells. Basal TNF‐α levels did not differ significantly among cows, sheep, and goats under control conditions. Exposure to PFOS alone slightly but significantly increased TNF‐α secretion in all three species compared to untreated controls (*p* < 0.05 to *p* < 0.01). GLY alone induced a stronger increase in TNF‐α production, particularly in sheep and goats, reaching statistical significance compared to both control and PFOS groups (*p* < 0.001). Co‐exposure to PFOS and GLY resulted in the highest TNF‐α concentrations across all species, with significant increases compared to single‐exposure groups (***p < 0.0001). Among species, sheep exhibited the most pronounced response, followed by goats and cows (Figure 3A). Control IL‐6 levels were low and comparable across species. PFOS exposure alone did not significantly alter IL‐6 levels, whereas GLY treatment led to a modest but significant increase in IL‐6 in goats and cows (*p* < 0.05). The combined PFOS + GLY treatment markedly elevated IL‐6 secretion in all species, with goats showing the strongest response, reaching nearly 4.5 pg/mL, significantly higher than all other conditions (***p < 0.0001). Overall, both PFOS and GLY stimulated proinflammatory cytokine release, with GLY exerting a stronger effect than PFOS (Figure 3B). Co‐exposure had a synergistic effect, particularly evident for IL‐6 in goats and TNF‐α in sheep.

**FIGURE 4:**
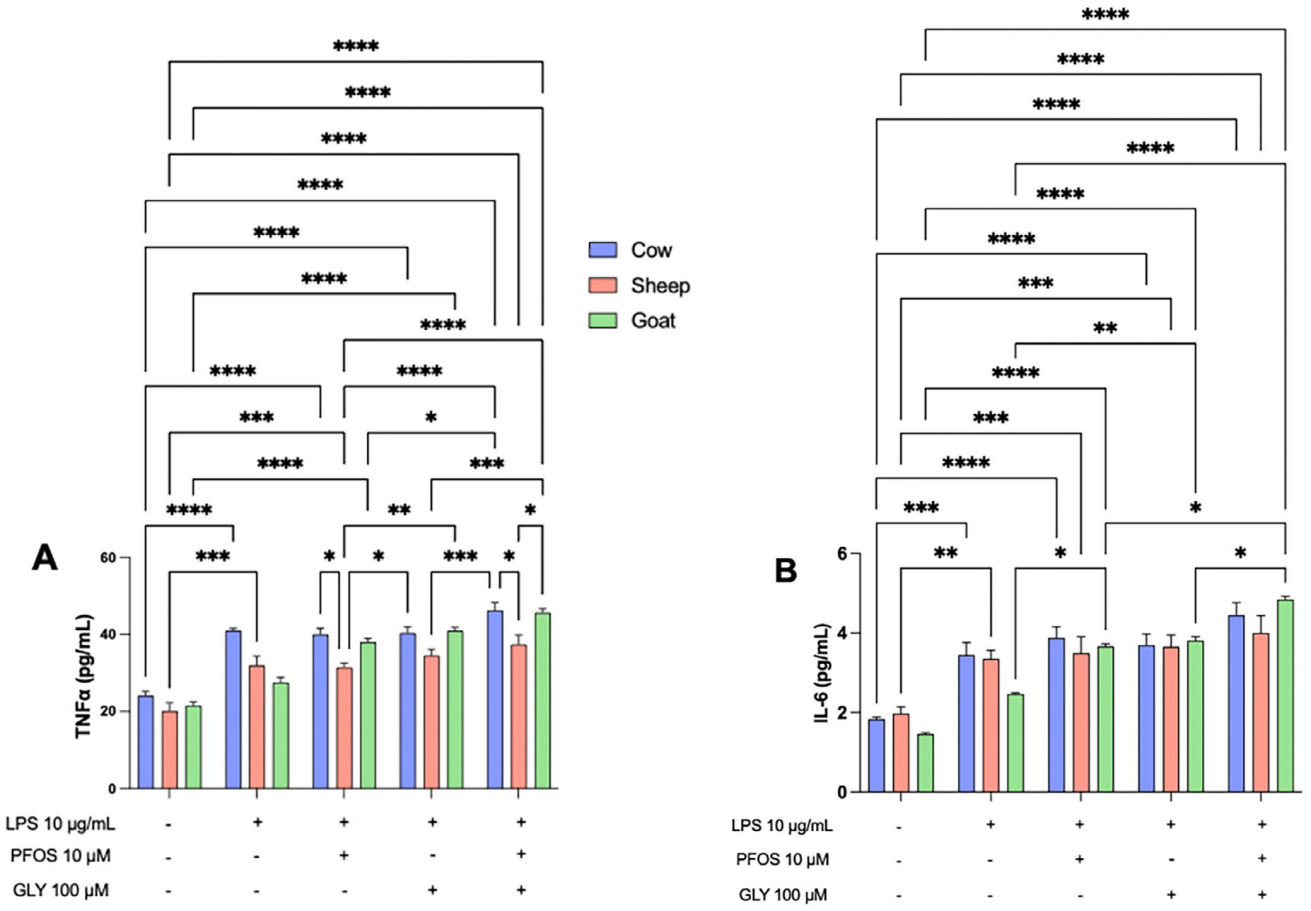
Graphs representing the results obtained in the evaluation of pro‐inflammatory cytokines TNF‐α (A), and IL‐6 (B). Data are representative of at least three experiments, means ± SEM. *p value < 0.05; **p value < 0.01; ***p value < 0.001; ****p value < 0.0001.

To assess the immunomodulatory impacts of PFOS and GLY when a general inflammatory stimulus is added, whole blood samples were exposed to LPS in the presence of the tested endocrine disruptors. Stimulation with LPS alone significantly increased TNF‐α secretion in all species compared to unstimulated controls (***p < 0.0001). Co‐treatment with LPS and PFOS further enhanced TNF‐α production in cows, sheep, and goats, with significant differences relative to LPS alone (*p < 0.01 to ***p < 0.0001). LPS + GLY treatment also elevated TNF‐α levels, particularly in goats, where the response was significantly higher than in LPS‐only groups (*p* < 0.05 to **p < 0.001). The strongest induction of TNF‐α was observed with the combined LPS + PFOS + GLY treatment, which produced significantly greater cytokine levels than any single‐ or dual‐treatment condition across all species (***p < 0.0001) (Figure 4A). As with TNF‐α, LPS markedly increased IL‐6 secretion compared to controls (***p < 0.0001). Addition of PFOS or GLY to LPS‐stimulated cells resulted in further elevation of IL‐6 levels, with goats again showing the most robust response. The combined exposure to LPS + PFOS + GLY significantly amplified IL‐6 secretion compared to LPS alone and dual treatments, reaching the highest cytokine levels in all species (***p < 0.0001) (Figure 4B). Taken together, these results demonstrate that PFOS and GLY synergistically potentiate LPS‐induced proinflammatory cytokine production. Although all three ruminant species showed enhanced responses, goats exhibited the most pronounced increases in both TNF‐α and IL‐6 secretion under combined exposure conditions.

## 4 Discussion

The results obtained in the present study provide a thorough investigation into the immunomodulatory effects of two omnipresent environmental pollutants—PFOS and GLY—at acute exposures in whole blood obtained from three ruminant mammal species of major economic and ecological importance ( *Bos taurus* , *Capra hircus* , *Ovis aries* ). This research was motivated by the central ecological role of grazing animals and their contribution to human food resources. The study demonstrated in detail how PFOS and GLY, alone and in combination, modulate key biomarkers of oxidative stress, inflammatory responses, and overall immune functions.

By employing an ex vivo exposure model, this work allowed precise control over contaminant concentrations, minimizing confounding variables inherent in in vivo studies. Plasma samples harvested after 24 h of incubation revealed significant increases in oxidative stress (MDA levels), nitric oxide production, and cytokine secretion (TNF‐α and IL‐6). Notably, sheep and goats displayed greater susceptibility to PFOS‐ and GLY‐induced oxidative stress and inflammatory activation compared to cows, underscoring the importance of interspecies variability in toxicological assessments [29, 32].

Crucially, cytokine measurements revealed a synergistic effect of PFOS and GLY co‐exposure, leading to a marked increase in TNF‐α and IL‐6 even in the absence of LPS stimulation. This indicates that the combined pollutants can induce a heightened basal pro‐inflammatory state, thereby disrupting immune homeostasis and potentially predisposing animals to dysregulated immune responses. These findings align with previous reports of pollutant‐driven immune activation [28, 33, 34] and add to growing evidence that PFOS and GLY can act in concert to amplify immune dysfunction [35, 36].

While the ex vivo approach is a powerful tool for dissecting direct contaminant effects, it is important to acknowledge its limitations. This model cannot fully recapitulate the complex pharmacokinetics, metabolic transformations, tissue distribution, and chronic adaptive responses occurring in a living organism [37, 38]. As such, extrapolation of these findings to long‐term in vivo scenarios should be made with caution. Future studies should incorporate chronic, low‐dose exposure models in animals to assess systemic and long‐term health impacts [30, 39].

The observed synergy between PFOS and GLY also raises important mechanistic questions. One plausible hypothesis is that PFOS and GLY act on distinct but convergent pathways in immune cells. PFOS, with its surfactant‐like properties, is known to integrate into and perturb cellular membranes, which could amplify receptor‐mediated signaling cascades, including those triggered by pro‐inflammatory stimuli [40]. GLY, on the other hand, has been implicated in mitochondrial dysfunction and oxidative stress, which can further activate inflammatory transcription factors such as NF‐κB [41]. The combination of membrane perturbation (by PFOS) and oxidative stress induction (by GLY) may therefore converge on shared signaling nodes, synergistically heightening cytokine release. Exploring such mechanistic interactions will provide deeper insights into pollutant‐induced immune dysregulation and guide targeted research [42].

In comparing our findings with previous work, several areas of convergence and divergence emerge. Consistent with earlier studies reporting PFOS‐induced cytokine release and GLY‐associated oxidative stress [25, 33, 34], our results confirm that both contaminants independently activate proinflammatory pathways. The observed synergistic elevation of TNF‐α and IL‐6 under co‐exposure also aligns with findings by Franco et al. [25], who demonstrated similar pollutant‐driven amplification of cytokine secretion in mammalian immune cells. However, our data diverge from reports indicating negligible or modest cytokine modulation by GLY alone [37], as we detected significant proinflammatory effects, particularly in goats. This discrepancy may reflect species‐specific susceptibility, differences in experimental models (whole blood versus isolated immune cells), or variations in GLY formulations. Moreover, while previous investigations have predominantly focused on single‐species or in vitro systems [38, 40, 41], our ex vivo cross‐species approach highlights interspecies variability, emphasizing the greater vulnerability of sheep and goats compared to cows. These differences underscore the necessity of using comparative models in immunotoxicology to fully capture the ecological and agricultural implications of contaminant exposure.

The ecological and human health implications of these findings are significant. Ruminants exposed to PFOS and GLY may experience impaired immunity, reduced productivity, and increased susceptibility to infections and environmental stressors. Given the bioaccumulative nature of PFOS and the widespread agricultural use of GLY, the transfer of residues through the food chain represents an underappreciated risk to human health [35, 36]. Thus, these findings reinforce the need for species‐specific risk assessment, stricter regulatory monitoring, and future research into chronic, low‐dose exposures and mechanistic underpinnings of pollutant synergy. These results represent the bases for further research to fully understand the complex molecular mechanisms underlying such immunomodulatory effects [39].

## Author Contributions

Investigation: F.M. and G.A.F. Formal analysis: A.I., F.I., and N.T. Writing – original draft preparation: M.R. Writing – review and editing: D.D.P. Conceptualization: E.G. Supervision: G.P. and D.D.P. All authors have read and agreed to the published version of the manuscript.

## Funding

The authors have nothing to report.

## Disclosure

No artificial intelligence tools were employed for data generation, data analysis, image creation, or image manipulation.

## Conflicts of Interest

The authors declare no conflicts of interest.

## Data Availability

The data that support the findings of this study are available from the corresponding author upon reasonable request.
